# A Novel Turn-On Fluorescent Sensor Based on Sulfur Quantum Dots and MnO_2_ Nanosheet Architectures for Detection of Hydrazine

**DOI:** 10.3390/nano12132207

**Published:** 2022-06-27

**Authors:** Xin Li, Xiaobin Wang, Wei Guo, Feng Luan, Chunyuan Tian, Xuming Zhuang, Lijun Zhao

**Affiliations:** 1 College of Chemistry and Chemical Engineering, Yantai University, Yantai 264005, China; lixinxin970519@163.com (X.L.); w1428566822@163.com (X.W.); luanf@ytu.edu.cn (F.L.); cytian@ytu.edu.cn (C.T.); 2 Shandong Dyne Marine Biopharmaceutical Co., Ltd., Weihai 264300, China

**Keywords:** sulfur quantum dots, MnO_2_ nanosheet, hydrazine, fluorescence probe

## Abstract

In this paper, the SQDs@MnO_2_ NS as the probe was applied to construct a novel “turn-on” fluorescent sensor for sensitive and selective detection of hydrazine (N_2_H_4_). Sulfur quantum dots (SQDs) and MnO_2_ nanosheets (MnO_2_ NS) were simply mixed, through the process of adsorption to prepare the architectures of SQDs@MnO_2_ NS. The fluorescent emissions of SQDs@MnO_2_ NS play a key role to indicate the state of the sensor. According to the inner filter effect (IFE) mechanism, the state of the sensor at the “off” position, or low emission, under the presence of MnO_2_ NS, is which the ultraviolet and visible spectrum overlaps with the fluorescence emission spectrum of SQDs. Under the optimal conditions, the emission was gradually recovered with the addition of the N_2_H_4_, since the N_2_H_4_ as a strong reductant could make the MnO_2_ NS converted into Mn^2+^, the state of the sensor at the “on”. Meanwhile, the fluorescent sensor possesses good selectivity and high sensitivity, and the detection concentration of N_2_H_4_ with a wide range from 0.1 µM to 10 mM with a detection limit of 0.072 µM. Furthermore, actual samples were successful in detecting certain implications, indicating that the fluorescent sensor possesses the potential application ability to monitor the N_2_H_4_ in the water.

## 1. Introduction

Hydrazine (N_2_H_4_) has attracted particular attention due to its strong reducibility and weak alkalinity in applications such as pesticides, pharmaceuticals, fuels, organic dyes, and so on [1,2]. Meanwhile, the toxicity and harm of N_2_H_4_ could not be neglected due to its water-solubility. It could damage the lungs, eyes, skin, and some system diseases when exposed to the N_2_H_4_ surroundings for an extended period of time [3,4]. Hence, the development of a facile and sensitive measure for N_2_H_4_ is considerable. In the past decades, many analytical methods have been reported, including chromatography, electrochemical, fluorescent, titrimetric, colorimetry, and mass spectrometry [5,6,7]. The fluorescent method is a powerful technique to detect N_2_H_4_, due to a comprehensive consideration of the factors including the low cost, simple operation, and rapid analysis.

The fluorescent method consists of constructing a fluorescent probe to observe the fluorescence intensity enhancement, or quenching, for the qualitative and quantitative analysis present of the targets. The fluorescent probe materials are commonly applied in the fluorescent sensor field similar to quantum dots (QDs) [8,9,10], organics [11,12], metal-organic framework [13,14,15], and metal nanoclusters [16,17]. Therein, the sulfur quantum dots (SQDs) is a novel and attention the QDs, which retain the advantage of the traditional optical performance of QDs while overcoming potential issues of the toxicity of the heavy metal QDs. Thus, it is widely applied in the fluorescent probes, biological sensors, and cell imaging fields [18,19,20]. Lei et al., take the one-pot to prepare the polyvinyl alcohol-capped SQDs as the fluorescent probe for detection of Fe^3+^ and temperature in cells [21].

Nowadays, the various probes of fluorescent are being investigated and developed to detect N_2_H_4_. Based on the aggregation caused quenching effect [22,23,24], aggregation-induced emission effect [25,26,27], the excited-state intramolecular proton-transfer effect [28,29,30], and photo-induced electron transfer [31,32], probes such as 5-(9-phenyl-9H-carbazol-3-yl)thiophene-2-carbaldehyde [22], salicylaldehyde Schiff’s base [25], p-TNS [28], and 5-chlorothiophene-2-carbonyl chloride [31]. Using these mechanisms to detect N_2_H_4_ method is relatively mature, with little room for growth. Therefore, we introduced the inner filter effect (IFE) mechanism to rapidly detect N_2_H_4_, which is the absorption of the excitation and/or emission light by the quencher (MnO_2_) leading to the intensity decrease [33].

**Figure 1:**
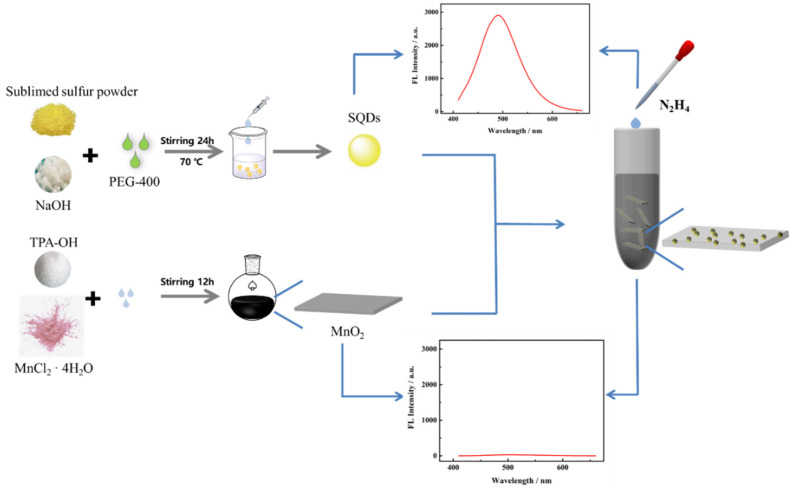
Mechanism of “turn-on” fluorescence sensor based on SQDs@MnO_2_ NS for detecting N_2_H_4_.

Herein, we first introduced the IFE mechanism to establish a “turn-on” fluorescent sensor for the detection of N_2_H_4_. The sensing strategy is illustrated in Figure 1; SQDs combined with MnO_2_ nanosheet (MnO_2_ NS) to prepare SQDs@MnO_2_ NS architectures. The SQDs alone have a strong fluorescence intensity and the MnO_2_ NS has nearly no fluorescence under the same experimental conditions. The SQDs@MnO_2_ NS possesses a lower intensity compared to the SQDs, due to the MnO_2_ NS as a full-of-all adsorbed material in the ultraviolet and visible (UV-Vis) spectrum, which could overlap with the fluorescence emission spectrum of SQDs, led to the fluorescence intensity quenching. Meanwhile, at this stage, the state of the fluorescent sensor is off. However, the emission of fluorescent is recovered under the N_2_H_4_ present condition, with the addition concentrations the state is gradually turned on. Benefits of the sensor for quantitatively detecting N_2_H_4_ was successfully constructed by monitoring the fluorescent intensity of SQDs@MnO_2_ NS. Furthermore, this approach possesses the potential for a practical application, due to its ability to effectively identify the N_2_H_4_ in the real samples of water.

## 2. Materials and Methods

### 2.1. Materials

Sublimed sulfur, polyethylene glycol (PEG-400), Tetramethylammonium hydroxide (TMA·OH), and NaOH were provided by Shanghai Aladdin Biochemical Technology Co. (Shanghai, China). MnCl_2_·4H_2_O, K_2_S_2_O_8_ were acquired from Sinopharm Chemical Reagent Co., Ltd. (Tianjin, China). N_2_H_4_ (*v*/*v* 80%) was purchased from Sigma Chemical Co., Ltd. (Shanghai, China). The prepared solutions of all experiments used ultrapure water (18.2 MΩ cm) from a water purification system.

### 2.2. Apparatus

Transmission electron microscopy (TEM) and high-resolution transmission electron microscopy (HR-TEM) measurements was carried out using a JEOL-2010F (200 kV) (JEOL, Tokyo, Japan). The ultraviolet and visible (UV-Vis) absorption spectra were examined with a UV-Vis spectrophotometer (TU-1901, Beijing, China). Fourier-transform infrared (FT-IR) spectroscopy was performed using a Nicolet 5700 Fourier transform infrared spectrometer (Shimadzu, Tokyo, Japan). The prepared nanomaterials were characterized by X-ray diffraction (XRD, LabX XRD-6000 (Shimadzu, Tokyo, Japan)). Elemental analysis was recorded by X-ray photoelectron spectroscopy (XPS, Thermo Scientific Escalab 250Xi, USA). Fluorescence spectra were collected using an F-4700 fluorescence spectrophotometer (HITACHI, Tokyo, Japan).

### 2.3. Synthesis of SQDs and MnO_2_ NS

SQDs were synthesized according to a literature method [34]. Briefly, the sublimed sulfur powder (1.4 g) was added to a mixed solution of PEG-400 (3 mL) and NaOH (50 mL, 0.08 g mL^−1^) stirring at 70 °C for 24 h. During the period, the color of the solution changed gradually from dark-yellow to light-yellow, and then added H_2_O_2_ (3 mL) to each, the obtained solution was termed as SQDs. The prepared SQDs were introduced in the dialysis membrane with the molecular weight of 1000 Da to remove unreacted molecular dialysis for 72 h each 12 h to change the water. Then, the light-yellow solid was acquired by freeze-drying at −20 °C for 24 h, and the SQDs were stored at 4 °C for further use.

MnO_2_ NS were prepared with reference to previous literature [35]. Firstly, TMA·OH (12 mL, 1.0 M) solution was introduced in MnCl_2_·4H_2_O (10 mL, 0.3 M) at the 50 mL round-bottomed flask. Afterward, the H_2_O_2_ (2 mL, 30%) solution was slowly added to the mixed solution vigorously stirring at room temperature for 24 h. The acquired dark brown solution was centrifuged and rinsed with ultra-water and CH_3_OH several times. Last, the obtained product of MnO_2_ NS was dried at room temperature.

### 2.4. The SQDs@MnO_2_ NS Fluorescent Probe Detection N_2_H_4_

The mixture solution of SQDs@MnO_2_ was obtained by SQDs and MnO_2_ NS mixed to stand for 1 h at room temperature. Next, the different concentrations of N_2_H_4_ solution (0.1 µM–10 mM) were added to the SQDs@MnO_2_ (1 mL) to react for 10 min at room temperature and perform fluorescence spectroscopy tests. Finally, a standard curve line was constructed between various concentrations of N_2_H_4_ and the recovery value of fluorescence intensity. In addition, the fluorescence probe selectivity, stability, and repeatability were studied under the optimal conditions.

### 2.5. Detection of Actual Samples

The fluorescence probe of SQDs@MnO_2_ NS was selected specifically for N_2_H_4_. To verify the performance in the actual sample of the probe, this was applied to detect the environmental water samples. Actual samples were acquired from the lake and river in Yantai. Briefly, the water samples were filtered with the 0.45 µm filter membrane to remove impurities. Then, to detect the N_2_H_4_ in the lake and river were used to prepare various concentrations of N_2_H_4_ (0.1 µM, 10 µM, and 10 mM) reaction for 10 min to test fluorescence spectroscopy, respectively. Three experiments were performed in parallel, and RSD was calculated.

## 3. Results

### 3.1. Characteristics of SQDs, MnO_2_ NS, SQDs@MnO_2_

**Figure 2:**
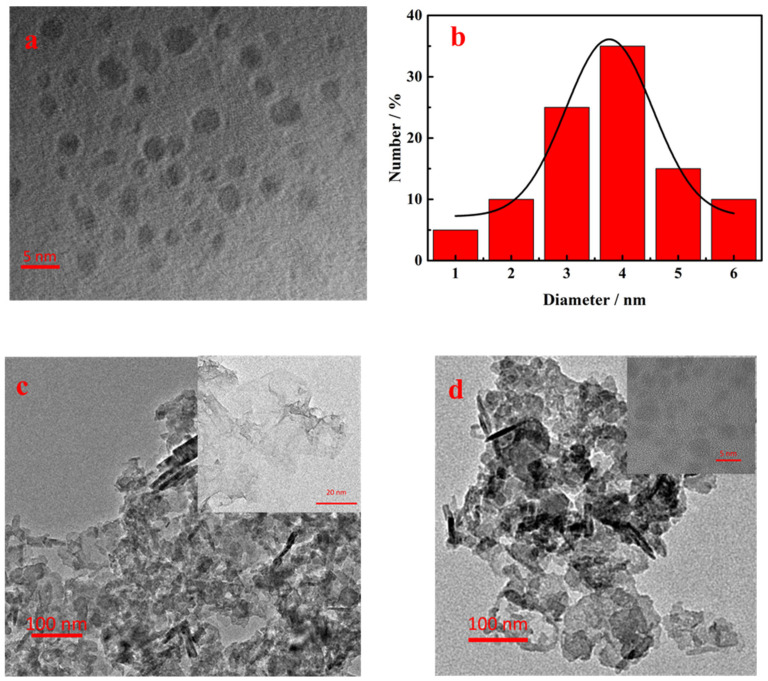
(**a**) HR-TEM images of SQDs; (**b**) the diameter distribution of the SQDs; (**c**) TEM images of MnO_2_ NS with HR-TEM images of MnO_2_ NS (inset); (**d**) TEM images of SQDs@MnO_2_ NS with HR-TEM images of SQDs@MnO_2_ NS (inset).

The morphology of SQDs, MnO_2_ NS, and SQDs@MnO_2_ architectures was characterized by HR-TEM and TEM. As shown in Figure 2a,b, the morphology of SQDs was spherical particles with good distribution, and the size of SQDs was calculated mainly to be 3.5 ± 0.5 nm. Next, the morphology of MnO_2_ NS was investigated presenting a large two-dimensional ultrathin planar structure (inset of Figure 2c). Meanwhile, the structure of MnO_2_ NS under the size of 100 nm of TEM appears to wrinkle and aggregation (Figure 2c). Additionally, as shown in Figure 2d, SQDs@MnO_2_ retained the planar structure but have a stronger aggregate phenomenon compared with MnO_2_ NS (Figure 2c), and the SQDs were distributed on the surface of MnO_2_ NS, indicating that the SQDs@MnO_2_ was successfully prepared.

**Figure 3:**
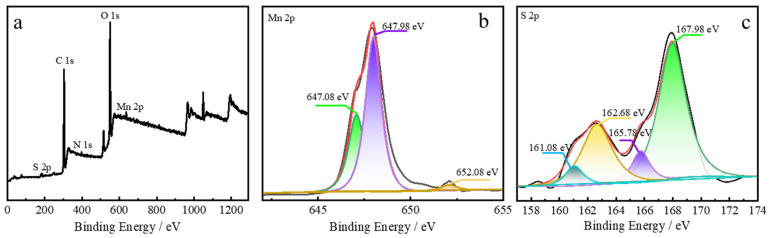
(**a**) XPS survey spectrum and (**b**) high-resolution Mn 2p, and (**c**) high-resolution S 2p XPS spectrum of SQDs@MnO_2_ NS.

To further study the elements of SQDs and MnO_2_ NS, X-ray photoelectron spectroscopy (XPS) was analyzed. In Figure S1a, the MnO_2_ NS was composed of four elements of C, O, N, and Mn. In the spectrogram of the Mn 2p element in Figure S1b, the band energy peaks located at 641.8 eV belonged to MnO_2_, and the characteristic peaks of Mn 2p appeared at 644.3 eV, 649.1 eV, which was identified with the previously reported work [36]. As can be seen in Figure S1c, the XPS survey spectrum of SQDs was recorded, which peaks corresponding to the elements of C, O, and S, respectively. The spectrum of the S 2p region in Figure S1d exhibits two peaks at 162.3 eV and 163.2 eV, which were due to the elemental S. The band peaks at 166.5 eV, 168.2 eV, and 169.3 eV were respective corresponding to the SO_3_^2−^ (2p_2/3_), SO_3_^2−^ (2p_2/3_) or SO_2_^2−^ (2p_1/2_), and SO_3_^2−^ (2p_1/2_), which demonstrated that the prepared SQDs the surface has an amount of sulfite group by adsorbing since the huge surface and small volume [34]. Additionally, the XPS survey spectrum of SQDs@MnO_2_ was shown in Figure 3a, in which elements of S 2p (Figure 3b) and Mn 2p (Figure 3c) correspond to the SQDs and MnO_2_, indicating the SQDs@MnO_2_ was successfully prepared.

**Figure 4:**
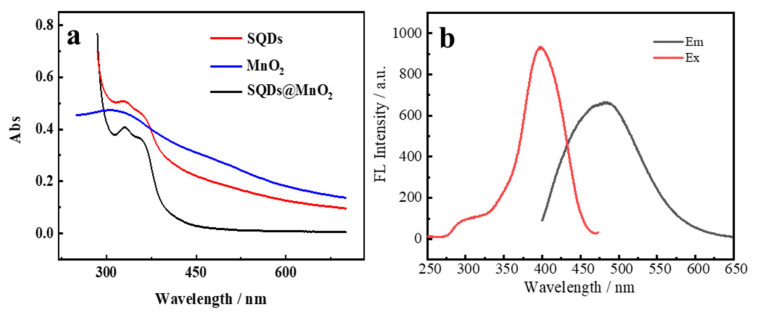
(**a**) UV-Vis spectra of SQDs, MnO_2_ NS, and SQDs@MnO_2_ NS; (**b**) the excitation and emission spectra of SQDs@MnO_2_.

To further verify the SQDs, MnO_2_ NS, and SQDs@MnO_2_ NS were successful in preparation, the UV-Vis spectra were shown in Figure 4. The broad absorption bands of MnO_2_ NS the range from 280 to 650 nm a weak peak around 360 nm, which is due to the d-d transition of Mn^4+^ ions [37]. The UV-Vis absorption spectra of SQDs and SQDs@MnO_2_ both have peaks at 313 nm and 350 nm, which might be ascribed to the S_2_^2−^ and S_8_^2−^ adsorbed on the surface of SQDs [34]. However, the values of peaks of SQDs@MnO_2_ were lower than SQDs due to the adsorption of SQDs on MnO_2_ NS. The excitation (Ex) and emission (Em) spectra of fluorescence of SQDs@MnO_2_ were shown in Figure 4b, the Em wavelength at 484.2 nm under the excitation wavelength of 380 nm, which is like the previous work [38].

### 3.2. Optimization of Experimental Parameters

**Figure 5:**
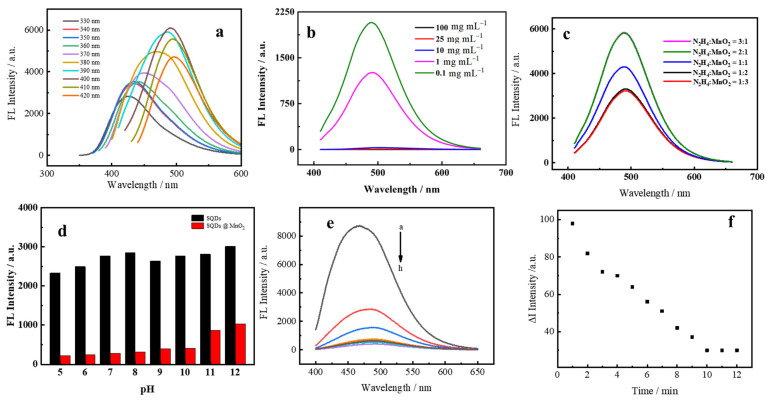
Optimization of conditions: (**a**) optimal excitation wavelength for SQDs; (**b**) concentrations of MnO_2_ NS; (**c**) volume ratio of N_2_H_4_ to MnO_2_ NS; (**d**) Different pH; (**e**) the molar ratio of SQDs@MnO_2_ (a–h (SQDs: MnO_2_ = 10:0, 10:1, 10:2, 10:3, 10:4, 10:5, 10:6, 10:7)); (**f**) the response time of SQDs@MnO_2_ with N_2_H_4_.

We have investigated the experimental parameters to acquire the optimal conditions, including the excitation wavelength for SQDs, the concentration of MnO_2_ NS, the volume ratio of N_2_H_4_ to MnO_2_ NS, and the pH of the SQDs and SQDs@MnO_2_ NS solution. As illustrated in Figure 5a, the synthesized of SQDs detected under the different excitation wavelengths at 330–420 nm, the intensity of fluorescent behaved a general trend of rising first and then falling, and the maximum emission at 400 nm. Thus, the excitation wavelength of SQDs at 400 nm was chosen as the optimal wavelength. As shown in Figure 5b, with the increase of the concentration of MnO_2_ NS, the quenching emission values of SQDs were increased, and the fluorescent intensity of SQDs was nearly all the quenched at the concentration of MnO_2_ NS at 10 mg mL^−1^. Hence, 10 mg mL^−1^ was selected as the optimum concentration of MnO_2_ NS for the next use. In addition, the quenching behavior of SQDs@MnO_2_ about different concentrations of MnO_2_ NS for better visualization in Figure S2, which obviously noted that the MnO_2_ NS possesses a huge surface that could package the SQDs. The volume ratio of N_2_H_4_ to MnO_2_ NS was shown in Figure 5c, the N_2_H_4_ volume-specific gravity increased the emission was gradually recovered, and the volume ratio reached 2:1 of N_2_H_4_ to MnO_2_ NS the emission intensity reached the maximum recovery values. Furthermore, the SQDs increased with pH from 5 to 12, which had no influence on its emission, while introducing the MnO_2_ NS the emission of SQDs values significantly decreased (Figure 5d). However, with the increased pH, the quench of emission degree was decreased. On this basis, we selected the pH = 7 as the experiment condition, considering the pH of the environment water. As shown in Figure 5e, the fluorescence of SQDs intensity was decreasing when the MnO_2_ was added. The molar ratio of SQDs@MnO_2_ was increased to 10:4 the fluorescence intensity reached its lowest. After, the molar ratio of SQDs@MnO_2_ over 10:4 the fluorescence intensity was a tiny increase. Thus, the molar ratio of 10:4 has been chosen for the further experiment. In addition, the response time of SQDs@MnO_2_ with N_2_H_4_ was recorded in Figure 5f, when 10 min of reaction was the ∆I = 30 (∆I = intensity (2 min)-intensity (1 min)), and the value of ∆I was nearly stable. Therefore, the SQDs@MnO_2_ with N_2_H_4_ 10 min of reaction as the optimal react time.

### 3.3. Fluorescence Spectra Analysis of N_2_H_4_ Sensing

The MnO_2_ NS nearly a total absorption in UV-Vi’s spectrum at the 280 nm to 650 nm in this study, which could effectively quench the fluorescence of SQDs due to the IFE mechanism. However, with the N_2_H_4_ was introduced once the emission was recovered, demonstrating that the MnO_2_ NS was reduced to Mn^2+^ in the presence of N_2_H_4_. Beneficial from this result, a simply “turn-on” sensor was constructed.

**Figure 6:**
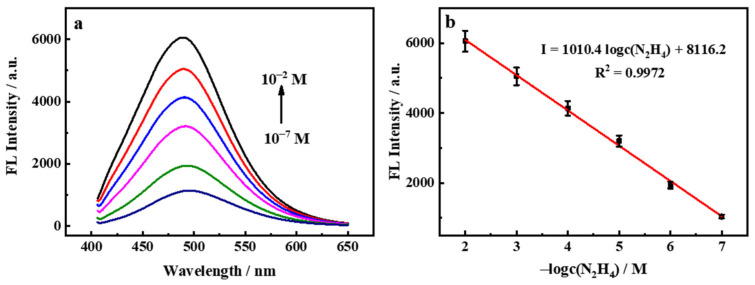
(**a**) The response of SQDs@MnO_2_ NS fluorescent probe to N_2_H_4_ solution with different concentrations (0.1 µM–10 mM); (**b**) The linear relationship between fluorescence intensity and N_2_H_4_ concentrations.

Under the optimum experiment condition, the analytical performance of the fluorescent sensor was investigated to detect N_2_H_4_ with various concentrations. As exhibited in Figure 6a, the fluorescence intensity was increased with the N_2_H_4_ concentration gradually added, indicating that the more reduction matter the more Mn^2+^ in the detected solution. The recovery values of fluorescence intensity of the logarithm of N_2_H_4_ concentration in the range from 0.1 µM to 10 mM, with a limit of detection (LOD) were calculated to be 0.072 µM according to the 3σ/s. Figure 6b demonstrates that the linear equation was I = 1010.4 logc(N_2_H_4_) + 8116.2 with a correlation coefficient of 0.9972, where I was the recovery intensity value of fluorescence. The comparison of the proposed methods to detect N_2_H_4_ with previous reports was listed in Table 1. It was significantly observed that the SQDs@MnO_2_ NS probe possessed the lower LOD and satisfactory linear range over other approaches.

### 3.4. Selectivity, Stability, and Repeatability

**Figure 7:**
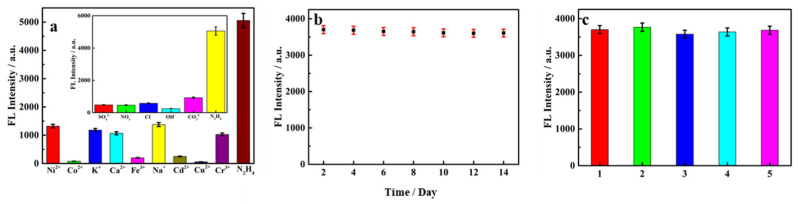
(**a**) The effect of different cations and anions on the fluorescence intensity of SQDs@MnO_2_ NS fluorescent probe; (**b**) the fluorescence intensity stability of SQDs in two weeks; (**c**) the reproducibility of SQDs in 5 groups.

To evaluate the specificity of the probe of SQDs@MnO_2_ NS, the selective as one of the most important factors was investigated under similar reaction conditions. The various ions including Ni^2+^, Co^2+^, K^+^, Ca^2+^, Fe^2+^, Na^+^, Cd^2+^, Cu^2+^, Cr^2+^, SO_4_^2−^, NO^3−^, Cl^−^, OH^−^, CO_3_^2−^ were used as interference agents, these ions are the common positive ions and anions present in the environment. As shown in Figure 7a, the fluorescence intensity was negligible present the interference agents compared to have N_2_H_4_, indicating that the preparation probe has a strong anti-interference ability and accuracy detect N_2_H_4_ in environment water.

In addition, to further assess the stability of the SQDs@MnO_2_ NS fluorescent probe, the good stability of SQDs was an important means to verify. As depicted in Figure 7b, the fluorescence intensity of SQDs was continuous detection for 14 days under similar experimental conditions, it was noticed that the intensity have a slow decrease and the degree was insignificant. Interestingly enough, after a month of observing the intensity of SQDs was only a tiny different compared with them before a month, illustrating that the SQDs@MnO_2_ NS possessed a high stable fluorescence performance. For reproducibility, as can be seen from Figure 7c, the test was performed under the five sets of parallel solutions of SQDs in the same environment, all of the measured fluorescence intensities possess the semblable value with an outstanding RSD of 1.1%. This result was successful in confirming that the SQDs have preeminent reproducibility. Meanwhile, they have the potential benefit to the synthesis and application of the SQDs@MnO_2_ NS. These results demonstrated that the proposed sensor has good selectivity, stability, and repeatability for the analysis of N_2_H_4_.

### 3.5. Detection of N_2_H_4_ in Real Water Samples

To investigate the practicability of the probe of SQDs@MnO_2_ NS, it was applied to detect N_2_H_4_ in real samples. Three parallel water samples were obtained from the local lake and river for conducting the standard recovery test. The results were shown in Table 2, the N_2_H_4_ was detected in the lake, river, serum, and saliva, where the recovery ranged from 90.21% to 109.1%, and the RSD was 0.9% to 4.5%, demonstrating that the fluorescent probe possesses practicability with promise for future applications.

## 4. Conclusions

In summary, we have developed a “turn-on” fluorescent sensor based on the SQDs@MnO_2_ NS architectures for the detection of N_2_H_4_. The MnO_2_ NS has a broad absorption band of MnO_2_ NS at 280 to 650 nm, which could effectively quench the emission of fluorescence of SQDs, owing to the IFE mechanism. However, the fluorescent emission was recovered presenting the N_2_H_4_ analysis target with a concentration in the range of 0.1 µM to 10 mM, with a LOD of 0.072 µM. In addition, the fluorescent sensor was successfully applied in real samples indicating the SQDs@MnO_2_ NS probe was possess the potential ability to detect the N_2_H_4_ in the environmental water samples.

## Figures and Tables

**Table 1:** Comparison of several different methods for N_2_H_4_ detection.

Method	Linear Range (M)	Detection Limit (M)	Ref.
ZY8 ^a^	1.6 × 10^−7^–6.2 × 10^−5^	1.6 × 10^−7^	[39]
PBAS ^b^	0–2 × 10^−5^	4.1 × 10^−7^	[25]
CEFN ^c^	0–6 × 10^−5^	9.6 × 10^−8^	[40]
HBTM ^d^	0–1.4 × 10^−4^	2.9 × 10^−7^	[30]
SQDs@MnO_2_ NS	10^−7^–10^−2^	7.2 × 10^−8^	This work

^a^ 3-hydroxyflavone; ^b^ Salicylaldehyde Schiff’s bases; ^c^ nopinone; ^d^ 5-acetyl-2-hydroxybenzaldehyde and 2-aminothiophenol.

**Table 2:** Recoveries for detecting N_2_H_4_ in real samples (*n* = 3).

Sample	Added (M)	Found (M)	Recovery (%)	RSD (%)
	10^−2^	1.073 × 10^−2^	107.3	1.4
Lake water	10^−5^	0.9021 × 10^−5^	90.21	2.1
	10^−7^	0.9624 × 10^−7^	96.24	1.1
	10^−2^	1.091 × 10^−2^	109.1	2.2
River water	10^−5^	1.032 × 10^−5^	103.2	0.9
	10^−7^	0.9254 × 10^−7^	92.54	1.7
	10^−2^	0.9691 × 10^−2^	96.91	4.5
Serum	10^−5^	0.9967 × 10^−5^	99.67	1.9
	10^−7^	1.027 × 10^−7^	102.7	2.8
	10^−2^	0.9851 × 10^−2^	98.51	3.1
Saliva	10^−5^	0.9741 × 10^−5^	97.41	1.6
	10^−7^	1.016 × 10^−7^	101.6	2.9

## Data Availability

Not applicable.

## Supplementary Materials

The following supporting information can be downloaded at: https://www.mdpi.com/article/10.3390/nano12132207/s1, Figure S1: (a) XPS survey spectrum of MnO_2_ NS and SQDs; Figure S2: The quenching behavior of SQDs@MnO_2_ about the concentrations of MnO_2_ NS.
