# Supplementary material for: A Novel Turn-On Fluorescent Sensor Based on Sulfur Quantum Dots and MnO2 Nanosheet Architectures for Detection of Hydrazine
Source: Nanomaterials (Basel). 2022 Jun 27;12(13):2207. doi: 10.3390/nano12132207 (PMC9268641; doi:10.3390/nano12132207)
Supplement: Supplementary file 1 [file nanomaterials-12-02207-s001.zip › nanomaterials-1746225-supplementary.pdf]

nanomaterials

Supplementary Materials

A Novel Turn-On Fluorescent Sensor Based on Sulfur Quantum Dots and MnO_2 Nanosheet Architectures for Detection of Hydrazine

Xin Li [1], Xiaobin Wang [1], Wei Guo [2,*], Feng Luan [1], Chunyuan Tian [1], Xuming Zhuang [1,*], Lijun Zhao [1,*]

1 College of Chemistry and Chemical Engineering, Yantai University, Yantai 264005, China; lixinxin970519@163.com (X.L.); w1428566822@163.com (X.W.); luanf@ytu.edu.cn (F.L.); cytian@ytu.edu.cn (C.T.)
2 Shandong Dyne Marine Biopharmaceutical Co., Ltd., Weihai 264300, China
* Correspondence: guodawei0298@163.com (W.G.); xmzhuang@iccas.ac.cn (X.Z.); zhaoljytu@ytu.edu.cn (L.Z.)

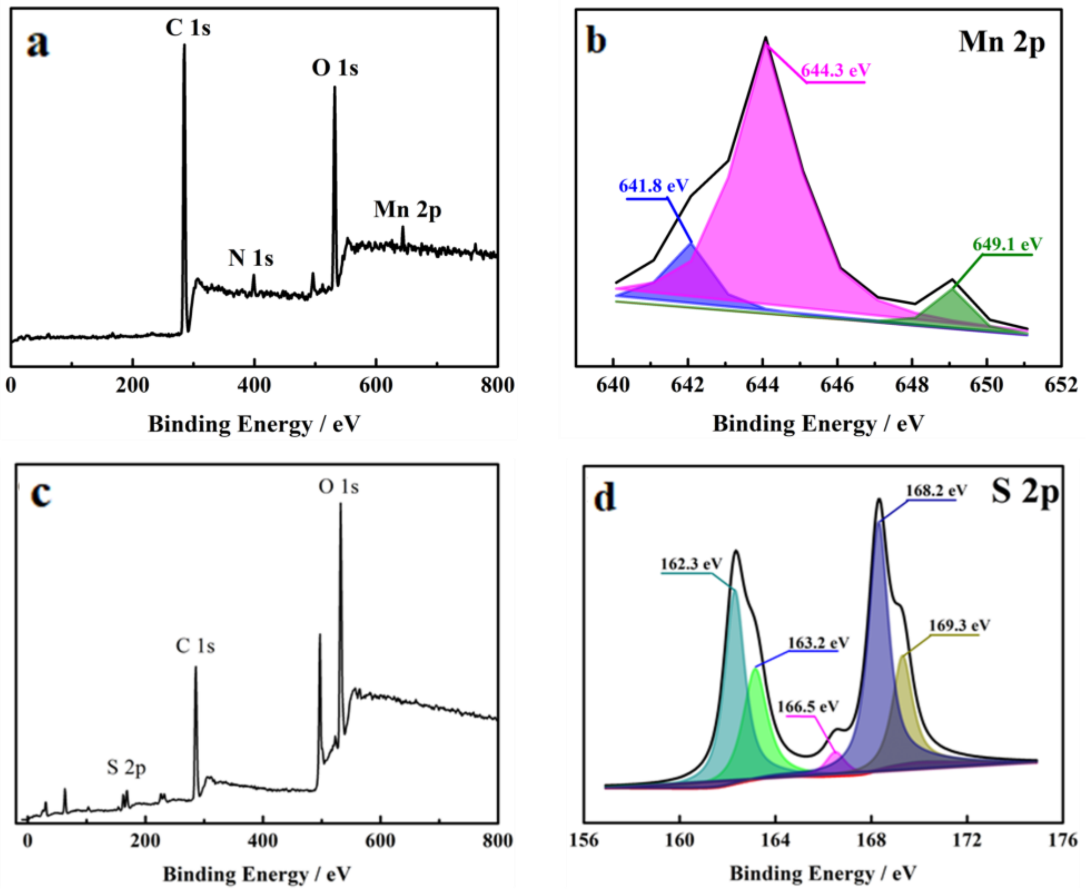

Figure S1. (**a**) XPS survey spectrum and (**b**) high-resolution Mn 2p XPS spectrum of MnO_2 NS. (**c**) XPS survey spectrum and (**d**) high-resolution S 2p XPS spectrum of SQDs.

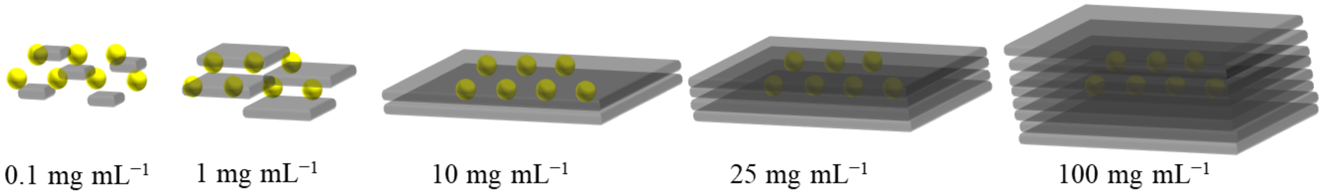

Figure S2. The quenching behavior of SQDs@MnO_2 about the concentrations of MnO_2 NS.
